# Modeling gut neuro-epithelial connections in a novel microfluidic device

**DOI:** 10.1038/s41378-023-00615-y

**Published:** 2023-11-14

**Authors:** Jose M. de Hoyos-Vega, Xi Yu, Alan M. Gonzalez-Suarez, Sisi Chen, Arnaldo Mercado-Perez, Eugene Krueger, Jeric Hernandez, Yaroslav Fedyshyn, Brooke R. Druliner, David R. Linden, Arthur Beyder, Alexander Revzin

**Affiliations:** 1 Department of Physiology and Biomedical Engineering, Mayo Clinic, Rochester, MN USA; 2 Enteric Neuroscience Program (ENSP), Mayo Clinic, Rochester, MN USA; 3 Division of Gastroenterology and Hepatology, Department of Medicine, Mayo Clinic, Rochester, MN USA

**Keywords:** Microfluidics, Nanostructures

## Abstract

The intestinal lumen is filled with diverse chemical and physical stimuli. Intestinal epithelial cells sense these stimuli and signal to enteric neurons which coordinate a range of physiologic processes required for normal digestive tract function. Yet, the neuro-epithelial connections remain poorly resolved, in part because the tools for orchestrating interactions between these cellular compartments are lacking. We describe the development of a two-compartment microfluidic device for co-culturing enteric neurons with intestinal epithelial cells. The device contains epithelial and neuronal compartments connected by microgrooves. The epithelial compartment was designed for cell seeding via injection and confinement of intestinal epithelial cells derived from human intestinal organoids. We demonstrated that organoids planarized effectively and retained epithelial phenotype for over a week. In the second chamber we dissociated and cultured intestinal myenteric neurons including intrinsic primary afferent neurons (IPANs) from transgenic mice that expressed the fluorescent protein tdTomato. IPANs extended projections into microgrooves, surrounded and frequently made contacts with epithelial cells. The density and directionality of neuronal projections were enhanced by the presence of epithelial cells in the adjacent compartment. Our microfluidic device represents a platform that may, in the future, be used to dissect structure and function of neuro-epithelial connections in the gut and other organs (skin, lung, bladder, and others) in health and disease.

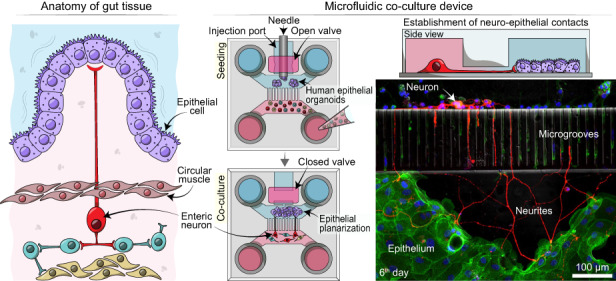

## Introduction

Epithelia are made up of a diversity of cell types. In the GI tract, all epithelial cells contribute to the barrier [1–4], while specialized sensory cells called enteroendocrine cells (EECs) sense chemical [5] and mechanical [6] stimuli in the luminal space. Also common to epithelia-covered organs is the communication between epithelia and sensory neurons via neuro-epithelial connections. These connections underlie all of the major senses, including touch, taste, smell, hearing and sight. In the internal organs, the neuro-epithelial connections form the basis of *interception* [7, 8]. The gut is unique among the interoceptive organs because in addition to extrinsic neuronal innervation, it has an expansive intrinsic nervous system, affectionately known as the “second brain.” Both extrinsic and intrinsic (enteric) sensory neurons form neuro-epithelial connections. Intriguingly, the gut’s intrinsic primary afferent (sensory) neurons (IPANs) are genetically similar to the extrinsic primary afferent neurons (ExPANs) [9]. The intrinsic neuro-epithelial sensory system in the gut involves epithelial sensing of luminal stimuli and relaying them to IPANs, which trigger a range of physiologic effects, such as control of vascular and intestinal smooth muscle and epithelial secretion, that are critical for proper digestion. Therefore, sensing luminal signals by the GI epithelium and its communication to enteric neurons are important elements contributing to GI function [10].

**Fig. 1:**
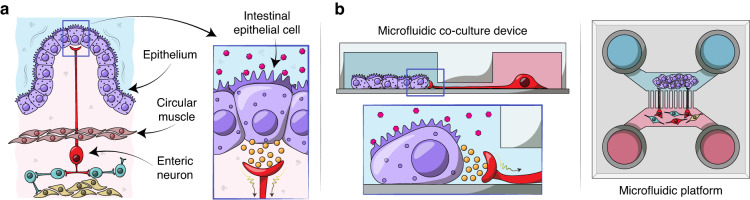
Creating enteric neuro-epithelial co-cultures in a microfluidic device. **a** Intestinal epithelial cells are located on the luminal lining of the gut. Upon receiving physical or chemical stimuli, intestinal epithelial cells communicate with enteric neurons such as IPANs in the myenteric plexus which is located between the circular and longitudinal layers of muscularis externa of the gut. Neuro-epithelial communications trigger expansion or contraction of the muscle layers in the gut. **b** Our microfluidic culture system was designed to recapitulate aspects of the neuro-epithelial interactions observed in vivo. This culture platform allowed to define and visualize neuro-epithelial contacts

Neuro-epithelial interactions have been challenging to study both in vivo and in vitro. Characterization in vivo is confounded by complex tissue anatomies – innervation complexity and large distances between the soma of innervating neurons and their target epithelia, high turnover and migration of epithelial cells, and active motility. Given the epithelia’s rapid turnover and the relative stability of neurons, culture conditions for these two populations differ dramatically. Epithelial cells have been cultured alone or co-cultured with neurons in vitro [11–14]. However, these preparations require the use of culture conditions (media and substrate functionalization) that favor either epithelia or neurons, affecting their functions. In addition, the random nature of neuro-epithelial cultures make it challenging to study interactions between the two cellular compartments. Our goal was to develop a culture system for placing intestinal epithelial and neuronal cells in distinct but proximal compartments to define and monitor neuro-epithelial contacts (see Fig. 1).

Microfluidic devices have been used extensively for cultivation of mammalian cells. Some of the early examples included hepatocytes alone or together with stromal cells where microfluidic perfusion was used to supply oxygen and nutrients [15, 16]. Subsequent work included cultures of intestinal epithelial cells within microfluidic devices providing shear and cyclical strain to stimulate and maintain phenotype of the cells [17–19]. The complexity of microfluidic cultures has progressed from cell lines (e.g., Caco-2) [20, 21] to primary cells (organoids) [22, 23] to incorporation of immune and microbial components along with oxygen gradients [24–27].

Microfluidic devices have also been used extensively for cultivation and studies of connectivity of neurons and glia [28, 29]. Such microfluidic devices are typically comprised of two or more cell culture compartments connected by microgrooves that are only large enough for neuronal extensions (axons or dendrites) but not soma to pass through [28, 30, 31], which enabled studies on neuronal synapses and interactions between different populations of neurons and between neurons and glia [32–34].

There is an increasing interest to intergate gut epithelium and neurons into microphysiological systems to study how gut-brain interactions contribute to disease pathogenesis and progression. These studies have typically utilized cellular models of gut and brain cultured in separate micro- or meso-fluidic modules connected by tubing or microchanels and have focused on the effects of microbial or epithelial secreted factors on the brain [35–38]. However, studies of neuro-epithelial connections that form and maintain over time in microphysiological systems that allow visualization are lacking.

Our paper describes the development of a microfluidic device for co-cultivation of intestinal epithelial cells and enteric neurons. As shown in Fig. 1b, the device contained two microfluidic compartments interconnected by microgrooves that guided neuronal processes into the epithelil compartment. The novel features of the device included: 1) a valve-controlled port to allow injection of intestinal organoid fragments directly into the cell culture chamber to minimize usage of scarce primary cells, 2) microgrooves designed to guide enetric neurites into the epithelial compartment and 3) pillars to retain organoid fragments and promote their planarization in the region adjacent to microgrooves. The microfluidic device allowed to co-culture primary intestinal epithelial cells and enteric neurons for 6 days while observing interactions between the two cellular compartments.

## Materials and methods

### Reagents

The following reagents were purchased from Sigma-Aldrich: propylene glycol monomethyl ether acetate (PGMEA, 484431), chlorotrimethylsilane (386529), poly-L-ornithine hydrobromide (P3655), 3-aminopropyl)triethoxysilane (APTES; 440140), SB202190 (S7067), Corning® Matrigel® growth factor reduced product (356231), acetylcysteine (NAC; 1009005), nicotinamide (N0636), gastrin I (05-23-2301) and Triton™ X-100 (X100). The following reagents were purchased from Thermo Fischer Scientific: bis-(sulfosuccinimidyl) suberate (Bs^3^; 21580), ethylenediaminetetraacetic acid, (EDTA; J15694.AE), Neurobasal A Medium (10888022), GlutaMAX (35050061), Advanced Dulbecco’s Modified Eagle Medium (ADMEM; 12491015), N2 supplement 100X (17502048), B27 supplement 50X (A1486701), Antibiotic/Antimycotic 100X (MT30004CI), TrypLE™ express enzyme 1X (12604021), and LIVE/DEAD™ viability/cytotoxicity kit (L3224). Rat collagen I, lower viscosity (3 mg/mL, 3443100-01) and epidermal growth factor (EGF, 236-EG) were purchased from R&D Systems. TGF-β1 inhibitor (A83-01; 2939/10) was purchased from Tocris and Y-27632 (A3008) from APExBio. Primocin (ant-pm-1) was purchased from Invivogen. Polystyrene beads of 16.3 (SVP-150-4) and 101 µm (SVP-1000-4) were purchased from Spherotech Inc. Sylgard 184 poly(dimethylsiloxane) (PDMS) kit (2065622) was purchased from Ellsworth and all SU-8 resists (2002, 2025, 2050, 2100) from Kayaku Advanced Materials.

### Fabrication of microfluidic devices

#### Mold fabrication

Two master molds were fabricated by photolithography, one for the flow layer (cell culture compartments, media transport channels and media reservoirs) and one for the valve layer. The design was done using CAD software (AutoCAD 2019, Autodesk Inc.) and exported as single files for each layer. The mold for the flow layer was fabricated on a 4-inch silicon wafer by sequential spin-coating and UV exposure of four SU-8 photoresist layers. 1) 50 µm tall alignment marks were fabricated using SU-8 2025. 2) 2.5 µm tall microgroove features were fabricated using SU-8 2002. 3) 100 µm tall features for neuronal compartment and transport channel were fabricated using SU-8 2050. 4) 300 µm tall features for the epithelial compartment and transport channel were fabricated using SU-8 2100. Each of the four steps involved spin-coating of resist, soft exposure bake, UV exposure, post exposure bake, developing and hard bake steps that were performed following the manufacturer’s instructions for each type of SU-8 resist. Exposure of all SU-8 layers was performed using a Micro Pattern Generator (µPG 101, Heidelberg, Germany). The valve mold was fabricated on a separate 4-inch silicon wafer and consisted of a single layer of SU-8 2100 resist with 400 μm tall features. The fabrication of this layer followed a sequence of steps described above and was accomplished with the same UV exposure tool. After both molds were fabricated, they were exposed to chloro-trimethylsilane in a closed chamber for ~30 min. The molds were then kept on a Petri dish until use.

#### Assembly of microfluidic devices

Microfluidic devices were fabricated using multilayer soft lithography. Briefly, the microfluidic device was composed of two layers of PDMS: flow and valve layer. The flow layer (bottom) was composed of two parallel cell culture compartments interconnected by microgrooves. The epithelial compartment contained a semicircle array of pillars and an injection port. The valve PDMS layer (top) was designed to place a valve above the injection port. A 20:1 and 5:1 wt/wt ratio of PDMS-curing agent was poured on the flow and valve mold, respectively; degassed for 30 min, and partially cured at 80 °C for 18 min. Afterwards, the PDMS valve layer was detached, cut, and the 0.5 mm inlet of the valve was punched. The valve PDMS slab was aligned on the flow layer and further baked at 80 °C for 2 h to promote bonding between the two layers. Next, the assembly was peeled off and inlets of the neuronal chamber were punched using 14 Ga needles. The inlets for the epithelial compartments were created using a 3 mm diameter puncher. Two strips of invisible tape (2 mm × 7 mm) were placed along the injection port and on the surface of a previously cleaned cover glass to protect the region of the valve during oxygen plasma treatment [39]. The PDMS assembly and the cover glass were exposed to oxygen plasma at 30 W for 3 min. The tape strips were removed from the assembly and the coverglass for alignment and bonding. Two 8 mm (d) × 8 mm (h) Pyrex cloning cylinders were bonded with uncured 10:1 PDMS mix on the neuronal chamber inlets, meanwhile two 10 mm (d) × 10 mm (h) cylinders were secured at the epithelial chamber inlets. The devices were cured at 80 °C for 30 min.

### Functionalization of microfluidic devices prior to cell seeding

Neuronal and epithelial compartments were functionalized with poly-L-ornithine and collagen I, respectively, using a well-established protocol [40, 41]. Briefly, the channels and compartments of a microfluidic device were incubated with 2.5% APTES in 95% ethanol for 20 min followed by a quick wash with 99% ethanol before being dried with nitrogen gas and incubated at 80 °C for 1 h. This step was designed to remove water and promote formation of an aminosilane layer on the glass. Consequently, the chambers were filled with 10 mM Bs^3^ in 1x PBS and incubated for 1 h at room temperature (RT). Afterwards, a microfluidic device was washed with distillated water and dried with nitrogen gas. Subsequently, the epithelial and neuronal compartments were infused with 0.3 mg/ml of collagen type I and 0.5 mg/mL poly-L-ornithine, respectively, and incubated for 1 h. Bs^3^ is a homobifunctional crosslinker covalently linking amines on glass to the amino groups on proteins or polypeptides. After the functionalization step, the devices were washed with fresh 1x PBS, degassed for 1 h and UV-sterilized for 1 h prior to seeding cells.

### Diffusion characterization in the microfluidic device

Media reservoirs of microfluidic devices were filled with equal volumes of 1x PBS and mounted on an inverted fluorescence microscope (IX-83, Olympus) using 10× long distance objective for timelapse imaging. Prior imaging, the saline solution in the epithelial compartment was exchanged by FITC-Dextran (MW 4 kDa) at a concentration of 100 µM in 1x PBS. After levels of solution equilibrated in reservoirs, fluorescence images were acquired from the central region of the device every 10 min for 4.5 h. Fluorescence intensity analysis was performed using ImageJ.

### Culturing human colon organoids in Matrigel and seeding epithelial cells into microfluidic devices

Organoids were derived from histologically normal human colon biopsies or surgical resections under IRB 21-006244 at Mayo Clinic, Rochester, MN. Established procedures for the isolation of crypts were used to generate organoids used in this study [42–44]. Briefly, crypts were isolated from the biopsy or mucosa layer from fresh tissue by incubation in 5 mM EDTA at 4 °C for 60–75 min. The crypts were collected and embedded in ice-cold Matrigel domes on a 24-well plate and cultured in Human Colon (HC) media at 37 °C with 5% CO_2_. This media is based on ADMEM containing 50% Wnt, R-Spondin, and Noggin (WRN) from conditioned media of L-WRN cell line (ATCC) and supplemented with: N2 supplement (1X), B27 supplement (1X), EGF (40 ng/ml), SB202190 (3 μM), A83-01 (500 nM), Y-27632 (10 μM), NAC (1 μM), nicotinamide (10 mM, Sigma), gastrin I (10 nM), primocin (100 μg/ml), and antibiotic/antimycotic (1X). Colon organoids were passaged every 7–10 days, and for preparation of organoids into microfluidic devices, they were digested to small fragments or single cells with TrypLE for up 30 min at 37 °C and filtered using a 70 µm strainer (352350; Cardinal Health).

Prior to seeding into the microfluidic device, media was removed from reservoirs feeding epithelial compartments and a house vacuum line was connected to the microfluidic device for valve actuation (opening). 30 µL of HC media containing organoid fragments at ~3 × 10^5^ cell/ml concentration were gently aspirated using a 25 Ga needle connected via Tygon tubing (06419-05; Cole-Parmer) to 1 ml BD Luer-Lok syringe (30 9628, BD). The needle was then introduced into the injection port and 1–2 µL of cell suspension was released in the epithelial chamber. Afterwards, the needle was removed, and the device was disconnected from the vacuum which returned the valve to its normally closed state. The microfluidic device was incubated for ~1 h at 37 °C with 5% CO_2_ to ensure cell attachment. Then, 500 µL of HC media was added into one of the reservoirs feeding epithelial compartment to 1) flush away unattached cells and 2) supply media in a sufficient amount for cultivation. Devices with cells were maintained at 37 °C with 5% CO_2_ with daily media exchanges. At the end of culture, the intestinal epithelial cells were exposed to calcein, ethidium homodimer and Hoechst to assess cell viability. Live/Dead assay was used per manufacturer’s instructions.

### Neuronal isolation and seeding into a microfluidic device

All animal experiments were performed under the National Institutes of Health (NIH) guidelines for ethical care and use of laboratory animals with the approval of the Institutional Animal Care and Use Committee (IACUC) of Mayo Clinic, Rochester, MN. Primary cultures of the myenteric plexus of the mouse small intestines were derived from transgenic Avil-CreERT2::tdTomato mice. This strain was created by breeding Avil-CreERT2 mice (Jax 032027) [45] with B6.Cg-Gt(ROSA)26Sor^tm14(CAG-tdTomato^)Hze/J mice (Ai14; Jax 007914) [30, 46] to hemizygosity and homozygosity, respectively. Cells of the small intestine myenteric plexus were isolated from dissected external muscle layers of the small intestine, as previously described [9]. Briefly, tissues were incubated first in a solution of 0.5 U/ml Collagenase A (Roche, Cat# 70474031), 2.2 U/ml Neutral Protease (Worthington, Cat# NPR02), 2.5 μg/ml DNAse I (Sigma, Cat# DN-25), 0.7 μg/ml choline TEA (Sigma, Cat# C-7527), and 0.3 mg/ml BSA (Sigma, Cat# A7906) in HBSS to isolate intact myenteric ganglia from surrounding muscle. Suspensions were passed over a 200 μm nylon filter (Pluriselect, Cat# 43-50200-03) to capture ganglia, and subsequently incubated in a solution of 250 U/ml Collagenase Type I (Worthington, Cat# LS004196) and 4.4 U/ml Dispase II (Roche, Cat# 18538700) where they were triturated with polished glass pipettes.

Dissociated cells from two mice were pooled in a 15 ml Falcon tube, centrifuged, resuspended in 20 µL of Neurobasal A media in 1.5 mL Eppendorf tube and placed on ice. Neurobasal A media was supplemented with 2% B27, 1% GlutaMAX and Antibiotic/Antimycotic 100X (MT30004CI).

A microfluidic device was first primed with supplemented Neurobasal A media for 5 min at 37 °C. Then, the media was aspirated from the reservoirs feeding the neuronal compartments and replaced with 5 µL of neuronal cell suspension (1 × 10^6^ cell/mL). The device was then incubated for ~1 h at 37 °C to ensure cell attachment. Afterwards, the unattached cells were washed away from the cell compartment by adding 300 µL of fresh Neurobasal media into one media reservoir, creating a difference in hydrostatic pressure and driving media into the device. After aspirating media with unattached cells, 500 μL a of Neurobasal media supplemented with 5 µM A83-01 (TGF-β1 inhibitor) were placed into each media reservoir feeding the neuronal compartment. Devices with neuronal cells were cultured at 37 °C, 5% CO_2_ with daily exchanges of Neurobasal A media supplemented with 5 µM A83-01.

### Creating neuro-epithelial co-cultures in a microfluidic device

A microfluidic device was assembled and functionalized with cell-adhesion ligands as described in Sections 'Fabrication of microfluidic devices' and 'Functionalization of microfluidic devices prior to cell seeding', respectively. Epithelial cells derived from human colon organoids were seeded into the epithelial compartment as described in Section 'Diffusion characterization in the microfluidic device'. During seeding of epithelial cells, the neuronal side of the microfluidic device was filled with Neurobasal media (see supplementary Fig. S1). The epithelial cells were allowed to attach inside the device for 1 h, after which excess cells were washed away by filling one media reservoir with 300 μL of media and allowing media to equilibrate between the reservoirs. Neuronal cells were seeded 24 h after introduction of epithelial cells, according to the protocol described in Section 'Culturing human colon organoids in Matrigel and seeding epithelial cells into microfluidic devices'. The neuronal cells were incubated for 1 h, after which excess cells were washed away with Neurobasal media and cultured in Neurobasal media supplemented with 5 µM A83-01. Microfluidic cultures were maintained at 37 °C, 5% CO_2_ with daily media exchanges.

### Immunofluorescence staining of neuro-epithelial cultures

For immunofluorescence staining, microfluidic devices were washed with 1x PBS and perfused with 0.2% TritonX-100 for 2 min on ice. Subsequently, the chambers were washed with 1x PBS and incubated with 4% PFA for 20 min at RT. Afterwards, the devices were washed with 1x PBS and further permeabilized with 0.05% TritonX-100 for 5 min on ice. Cells were incubated for 1 h at RT with the following primary antibodies: goat anti-human zonula occludens (ZO)-1 (1:100; Invitrogen) and mouse anti-human e-cadherin (1:100; BD Biosciences). Afterwards, the chambers were washed with PBS and blocked with 1% BSA in 1x PBS for 1 h at RT. The devices were washed with 1x PBS and incubated for 1 h with following secondary antibodies: Alexa-488 donkey anti-mouse IgG (1:1000; Thermo Fisher Scientific), Alexa-647 donkey anti-goat IgG, (1:1000; Thermo Fisher Scientific). Cell nuclei were stained using 4,6-diamidino-2- phenylindole (DAPI)(1:1000; BD Biosciences). As the last step, the devices were washed with 1x PBS and filled with fresh 1x PBS. Micrographs were obtained with an inverted fluorescence microscope (IX-83, Olympus) using 10× and 20× long distance objectives. Images were analyzed using ImageJ.

### Live-cell imaging in microfluidic devices

Cells were cultured and imaged in the microfluidic device. Imaging was performed on a Zeiss LSM980 confocal laser scanning microscope (Carl Zeiss Microscopy, LLC, White Plains, NY) using a 40X, 1.2 numerical aperture (NA) water-immersion objective lens and equipped with stage-top incubation set to 37 °C and 5% CO_2_. Z-stacks were acquired every 2 h for 62 ho (32 frames total). Microscope control, post-acquisition image analysis, and 3D projections were done using Zen 3.4 (blue edition, Carl Zeiss Microscopy, LLC, White Plains, NY).

## Results and discussion

We describe the development and characterization of neuro-epithelial co-cultures assembled in a microfluidic device. Intestinal epithelial cells and enteric neurons were shown to maintain phenotype and make contacts in this microfluidic device.

### Design of the microfluidic device

**Fig. 2:**
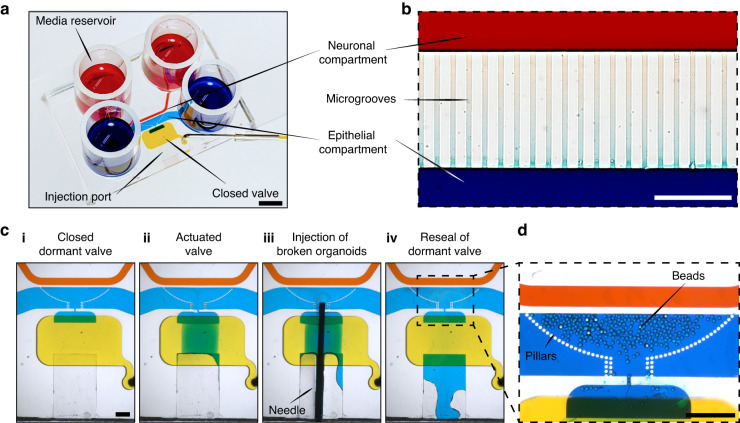
Microfluidic device for creating neuro-epithelial co-cultures. **a** An image of the microfluidic device. Neuronal and epithelial sides of the microfluidic device were filled with red and blue dye respectively. Yellow dye shows the normally closed valve. Cloning cylinders were used as media reservoirs. Scale bar: 5 mm. **b** Close-up view of the microgroove region located at the junction of neuronal and epithelial compartments. Scale bar: 100 µm. **c** A series of images describing the steps of valve opening, needle insertion, transfer of organoid pieces and re-sealing of the valve. **d** A close-up view showing that an array of posts helped retain polystyrene microbeads (100 μM diameter, approximating the size of organoid fragments) at the interface with neuronal compartment. Scale bar: 1 mm

The design criteria for the microfluidic device were to: 1) enable seeding and planarization of the intestinal organoids, 2) culture intestinal epithelial cells and enteric neurons in distinct but neighboring compartments, 3) guide the neuronal projections into the epithelial compartment and 4) enable visualization of the epithelial and neuronal compartments. The resulting microfluidic device is shown in Fig. 2a. It contained two cell culture compartments – larger compartment (4 cm by 1.5 mm and 300 μm in height) to be populated with epithelial cells derived from human colon organoids and a smaller compartment (4 cm by 0.5 mm with 100 μm height) for culturing enteric neurons. The two compartments were connected by microgrooves 150 μm in length and 2.5 μm in height. The concept of microgrooves was adapted from previously reported microfluidic devices designed for guiding axons between compartments while excluding soma. However, our microgrooves were made significantly shorter than those reported previously for CNS neurons (150 μm vs. 900 μm) to accommodate enteric neurites. (See Fig. 2b). The choice of microgroove dimensions was guided by several considerations. The neuronal compartment was made long and narrow to minimize the distance of neuronal soma from the grooves that allowed access to the epithelial compartment, thus increasing the chances of neuronal projections reaching the epithelial cells. The epithelial compartment was made taller to accommodate organoid fragments that ranged from 50 to 200 μm in size. Given that intestinal organoids were used as the source of epithelial cells, we wanted to ensure efficient seeding in the region of the device where neuro-epithelial connections were most likely to occur. To achieve this, we incorporated an injection port that allowed us to insert a needle and transfer organoid fragments directly into the epithelial compartment (see Fig. 2c) [39]. This injection port was protected by a normally closed valve. The valve was opened by applying a house vacuum and reverted to its default (closed) state when disconnected from the vacuum. In addition, posts were incorporated in a semicircular configuration around the area of epithelial cell injection (see Fig. 2c). The posts had a pitch of 30 μm and were used to retain organoid fragments within the epithelial seeding area and in proximity to microgrooves. The posts did not, however, interfere with diffusion of nutrients from the media reservoirs to the epithelial compartment. Media was delivered from reservoirs (cylinders) located at the inlet and outlet of each compartment (see Fig. 2a) and was exchanged every 24 h. Devices were designed with simplicity of use in mind. No lines for controlling the injection port/valve and no flow for delivering media were required during culture.

### Characterizing diffusion of molecules between epithelial and neuronal compartments

**Fig. 3:**
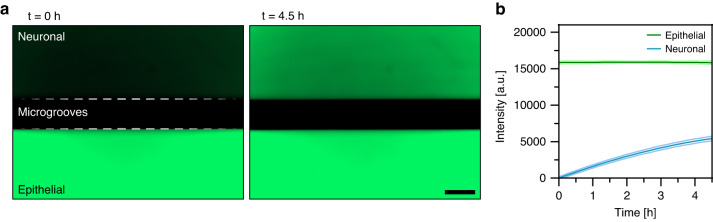
Characterizing diffusion between epithelial and neuronal microfluidic compartments. **a** Micrographs showing images of the microfluidic device at 0 and 4.5 h after injecting FITC-Dextran (4 kDa) in the epithelial compartment. Scale bar: 150 µm. **b** Changes in fluorescence intensity profiles for neuronal and epithelial compartments over the course of 4.5 h

As shown in Fig. 2b, our device contained microgrooves designed for guiding neuronal projections into the epithelial compartment. Such micro-constrictions have been used widely for axonal guidance in microfluidic devices [28]. We wanted to assess experimentally how presence of microgrooves affected the exchange of molecules between the two microfluidic compartments. To accommodate epithelial cell fragments from organoids the epithelial compartment was designed to be 8.7 times larger than the neuronal compartment (2.26 vs. 0.26 μL). Fluorescent dextran (MW 4 kDa) was used as a tracer molecule, was infused into the epithelial compartment, and its appearance in the neuronal compartment was monitored with fluorescence microscopy. As shown in Fig. 3a, after 4.5 h, the neuronal chamber reaches ~35% of the intensity compared to the epithelial chamber, suggesting that the timeframe to reach equilibrium is ~13 h. Diffusion was expected to also occur from neuronal to epithelial compartment, but was challenging to detect given the volume difference and dilution of signal. Overall, our characterization pointed to the existence of a robust exchange of signals between the two microfluidic compartments.

### Intestinal epithelial cultures in the microfluidic device

**Fig. 4:**
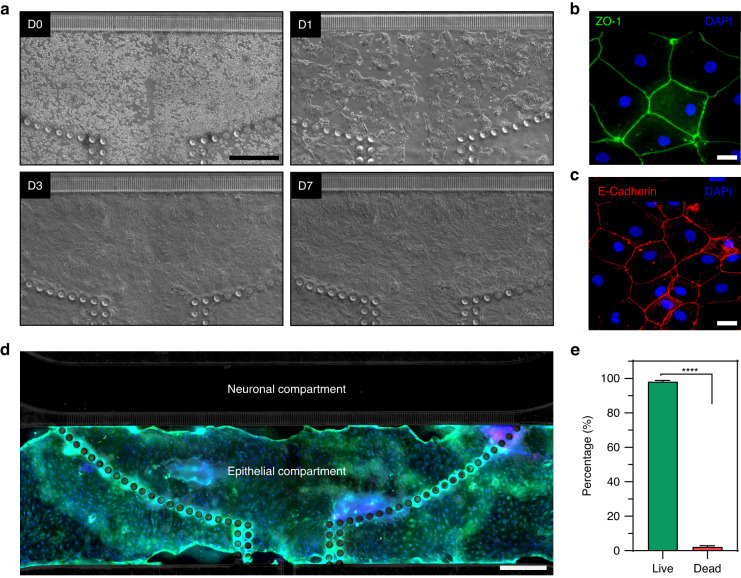
Formation of intestinal epithelial layer in the microfluidic device. **a** Micrographs showing epithelial cells at different timepoints during culture. Colon organoid fragments were injected into the device at day 0 (D0). The fragments formed patches 24 h after seeding (D1) that expanded, achieving 50–80% confluence at day 3 (D3) and 100% confluence by day 7 (D7). **b** Immunofluorescence of epithelial layer for ZO-1 (day5). **c** Immunofluorescence staining of epithelial layer for e-cadherin (day 5). **d** Viability staining after 10 days of culture where live and dead cells appear in green and red fluorescence, respectively. Cell nuclei were stained with Hoechst (blue). Scale bar: 500 µm. **e** Quantification of viability using live/dead images. Data are represented as means ± SD of 3 microfluidic devices. Statistical significance determined by one-tailed unpaired t-test, ****p-value < 0.0001]

Partially dissociated organoids were introduced into the microfluidic device through an injection port described in the preceding section and were evenly distributed within the attachment area defined by the array of posts (see Fig. 4a). Injecting directly into the culture chamber allowed for efficient use of scant primary intestinal organoids. Organoid fragments attached to the collagen-coated glass surface of the epithelial compartment 2 h after seeding and organized into small patches 24 h after seeding (see Fig. 4a). These patches expanded, with epithelial layer reaching 50 to 80% confluence after 4 days and full confluence after 7 days of culture. Similar dynamics of organoid fragments planarizing into patches and expanding into a confluent layer were observed with the neuronal compartment containing either HC (epithelial) or Neurobasal media (data not shown).

The epithelial compartment of the microfluidic device was characterized by immunofluorescence staining for zonulin (ZO-1) and E-cadherin, markers of well-differentiated interconnected intestinal epithelium. The results, shown in Fig. 4b, c, highlight that the epithelial layer in the microfluidic device had high levels of ZO-1 and e-cadherin expression and that cells exhibited cuboidal morphology typical of columnar epithelium [47]. Intestinal epithelial cells were confined to the epithelial compartment and did not cross over into the neuronal compartment during 7 days of culture (data not shown). The dynamics of organoid planarization and epithelial layer formation observed in our device were consistent with planarized and cultured colon organoids in gut-on-chip devices with apical and basolateral compartments [22, 48, 49].

The viability of cells within the microfluidic epithelial layer was assessed at day 10 of culture. As highlighted by Fig. 4d, the epithelial layer was contiguous and occupied an area that extended beyond the initial seeding area demarcated by posts. Live/dead staining showed that 98 to 99% of cells within the layer were viable (Fig. 4d). The results in Fig. 4 highlight our ability to planarize organoids and form intestinal epithelial layer inside the microfluidic device.

### Enteric neuronal cultures in the microfluidic device

**Fig. 5:**
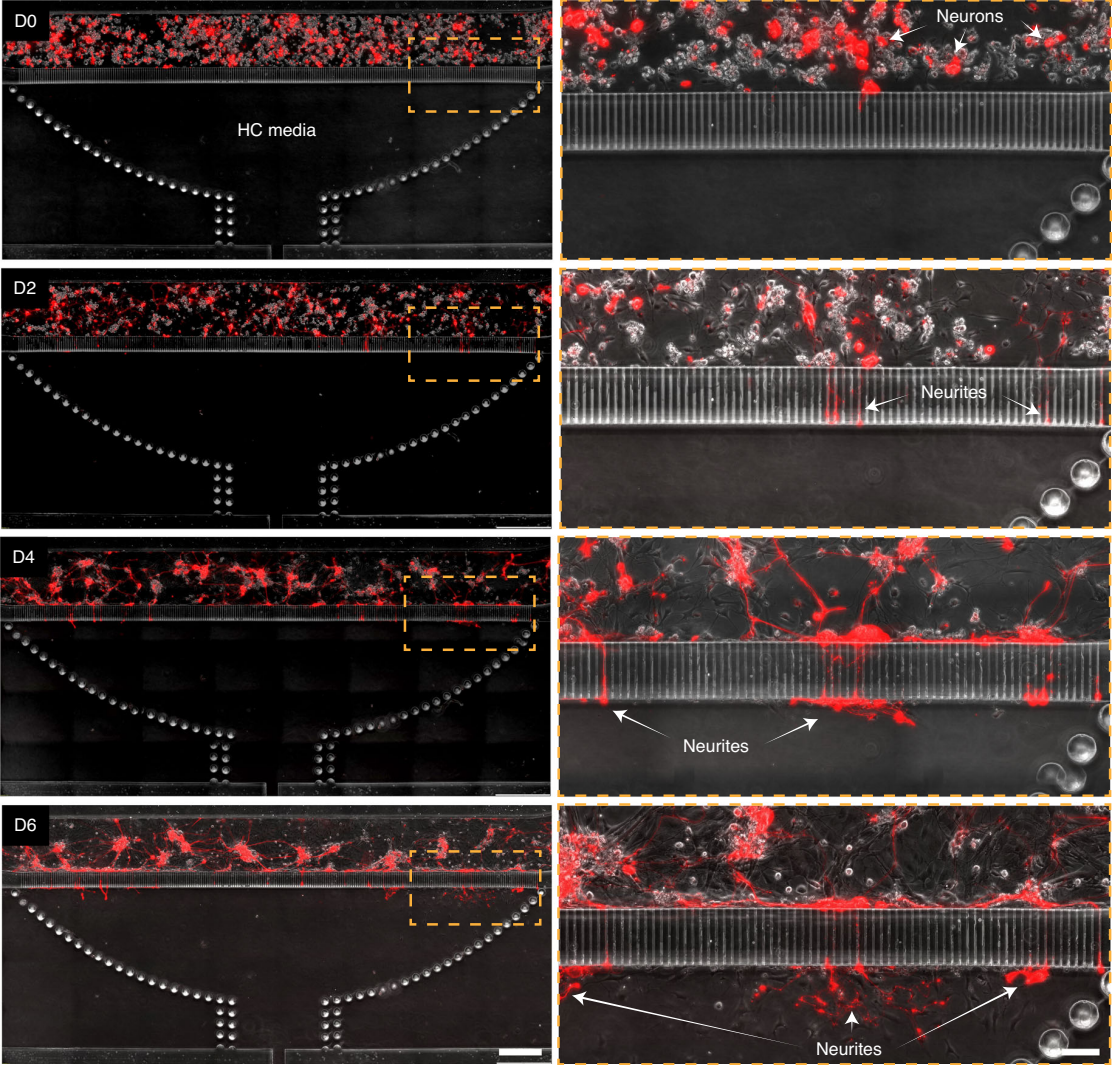
Enteric neurons cultured in the microfluidic device. A series of micrographs showing time-dependent changes in morphology of neuronal cultures. At day 0 (D0) IPANs (red) and non-fluorescent cells were injected into the chamber. The epithelial compartment was filled with epithelial HC media. After 48 h (D2), a complex network of neuronal processes formed in the compartment with some processes entering microgrooves. At day 4 (D4) and day 6(D6), neuronal processes exited microgrooves on the epithelial side but did not extend into the compartment. Scale bar: left column 500 µm, right column 100 µm

As the next step, we set out to assess enteric neuronal cell cultures in the microfluidic device. The cells were isolated from myenteric plexus and represented a mixture neurons ( ~ 8%) and glia ( ~ 75%), with some of the neurons being advillin+ tdTomato-expressing IPANs. As shown in Fig. 5, IPANs (red fluorescence) and other cells (no fluorescence) were uniformly distributed in the neuronal compartment shortly after seeding. Based on our previous work, mixed culture was deemed beneficial for maintaining healthy neurons in vitro [33]. However, we observed excessive proliferation of these likely non-neuronal cells in the microfluidic devices in some of the early experiments and supplemented the media with TGF-β inhibitor to remedy this [50, 51]. As seen from Fig. 5, the proportion of fluorescent IPANs and non-fluorescent cells remained constant over the course of 6 days in culture. As may be appreciated from higher magnification images, after about 4 days, the microfluidic neuronal cultures reassembled into clusters with features resembling a ganglionated plexus.

Neuronal cultures changed phenotype over time. A small number of neurons extended projections into the microgrooves 48 h after seeding (see Fig. 5). After 72 h of culture, neurons formed ganglion-like structures with a complex network of processes covering most of the neuronal compartment. At this time point, some neuronal projections entered the microgrooves, however, the projections frequently looped back into the neuronal chamber (see Fig. 5). This was independent of the culture media (neuronal or epithelial) present in the unseeded epithelial compartment.

We should also note that a small number of cells, likely glia and fibroblasts, was observed to infiltrate epithelial compartment. These cells proliferated and became noticeable after 5 days of culture (see Fig. 5). The likely reason for infiltration is relatively short (150 μm) microgrooves used by us to ensure the presence of neuronal extensions in the epithelial compartment. In the future, microgrooves may be made shallower, narrower and/or longer to minimize or eliminate cell infiltration from neuronal compartment. We should note however that current device provided a sufficient window of time ( ~ 3 to 4 days) for observing neuro-epithelial interactions in the absence of neuronal cell contamination in the epithelial compartment. These results are discussed in the next section.

### Creating neuro-epithelial co-cultures in the microfluidic device

**Fig. 6:**
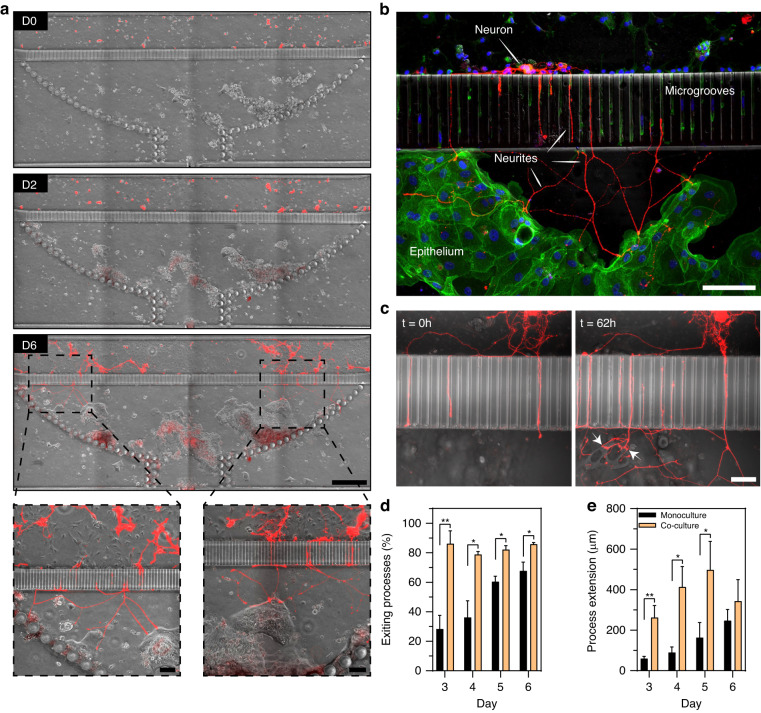
Neuro-epithelial co-cultures in the microfluidic device. **a** Time series of neuro-epithelial cultures over the course of 6 days. Epithelial monolayer patches were formed across the chamber and transgenic neurons (red) extended their process towards the epithelial compartment after 3 days of cultures. Scale bar: 500 µm. Scale bars in magnification images: 100 µm. **b** Confocal image of transgenic neuronal cells (red) extending process and making connectionswith epithelial cells stained with Vybrant DiO (green). Scale bar: 100 µm. **c** 3D image of neuro-epithelial connections in microfluidic device. White arrows indicate possible neuro-epithelial contacts. Scale bar: 50 µm. **d** Quantification of the percentage of neuronal processes exiting the grooves on the epithelial side. The number of neuronal processes in the epithelial compartment was divided by the total number of processes in the microgrooves to obtain % of exiting processes. **e** Quantification of the distance traveled by the neuronal processes in the epithelial chamber. During neuronal monocultures in (**d**) and (**e**), the epithelial compartment was filled with epithelial HC media. Data are represented as means ± SD of 4 microfluidic devices. Statistical significance determined by one-tailed unpaired t-test, *p-value < 0.05 and **p-value < 0.01

To create co-cultures, a microfluidic device was first populated with colon organoid fragments, which were allowed to acclimate for 24 h. At this point, fragments were attached (this process starts within 2 h of seeding) and began to form interconnected patches of epithelium (see Fig. 6a). Dissociated myenteric cells were seeded into the neuronal compartment of the device that already contained patches of epithelial cells. In the first two days of co-culture, we did not observe appreciable differences in the growth of neuronal processes compared to neuronal cells cultured alone. However, by day 3, IPAN neuronal processes were observed to traverse microgrooves, reach into the epithelial compartment, and make contact with epithelial cells. In days 4–6 of culture, numerous neuronal projections were observed in the epithelial compartment making connections with epithelial cells (see supplementary video 1). Interestingly, IPAN projections appeared to seek out epithelial patches as they were planarizing (Fig. 6a, D6), but epithelial cells also seemed to migrate toward neural processes and cause changes in those processes, including neurite pruning and redirection, upon contact (see supplementary video 1).

Fig. 6a shows a single epithelial cell innervated by multiple neuronal processes at day 6 of culture. In other instances, a single neuronal projection appeared to interact with multiple epithelial cells (see Fig. 6b). Some neuronal processes were observed to reach hundreds of micrometers into the epithelial compartment to make these connections.

We quantified the number and length of neuronal processes present in the epithelial compartment in the co-culture vs. mono-culture scenarios. The data, summarized in Fig. 6d, e, highlight that neuronal projections reached the epithelial compartment faster, in greater numbers and were longer in the presence of epithelial cells.

We note that epithelial compartment was filled with epithelial culture media for both monoculture and co-culture experiments. This means that paracrine signals of epithelial origin and not factors in present in the epithelial media were likely responsible for enhanced neuronal guidance observed in the co-culture format.

These results highlight our ability to use a novel microfluidic device for creating neuro-epithelial co-cultures where phenotype of an individual cell type was maintained and where enteric neurons innervated the intestinal epithelial layer.

## Conclusion

We designed and fabricated a novel microfluidic device for creating enteric neuro-epithelial co-cultures. To the best of our knowledge, this is the first demonstration of intestinal epithelial cells and enteric neurons co-cultured in a microfluidic device. We demonstrated that colon organoid fragments planarized in the device and formed a contiguous layer that was viable and retained markers of epithelium for at least 10 days. Enteric neuronal cells retained normal morphology and formed in vivo-like ganglion clusters in the microfluidic devices. Importantly, the microgrooves separating neuronal and epithelial compartments limited cells crossing over while allowing for neuronal projections to reach epithelium. Our microfluidic device was mounted on a glass cover slip that allowed for high-resolution time-lapse microscopy of neuro-epithelial interactions. We should note that our paper focuses on establishing a methodology to co-culture intestinal epithelial cells with enteric neurons and stops short of assessing the structure and function of contacts formed in these co-cultures. This assessment represents the next phase of our work. Overall, the microfluidic device described may, in the future, be used to improve understanding of disease mechanisms that underly functional gastrointestinal disorders, such as irritable bowel syndrome (IBS).

### Supplementary information

Supplemental material

Supplement Video 1:Formation of neuro-epithelial connections in microfluidic cultures
