# Supplementary material for: Modeling gut neuro-epithelial connections in a novel microfluidic device
Source: Microsyst Nanoeng. 2023 Nov 14;9:144. doi: 10.1038/s41378-023-00615-y (PMC10643697; doi:10.1038/s41378-023-00615-y)
Supplement: Supplementary file 1 — Supplemental material [file 41378_2023_615_MOESM1_ESM.docx]

**Modeling Gut Neuro-Epithelial Connections in a Novel Microfluidic Device**

José M. de Hoyos-Vega^1^, Xi Yu^1^, Alan M. Gonzalez-Suarez^1^, Sisi Chen^1^, Arnaldo Mercado-Perez^1^, Eugene Krueger^1^, Jeric Hernandez^1,2^, Yaroslav Fedyshyn^1^, Brooke R. Druliner^1,3*^, David R. Linden^1,2*^, Arthur Beyder^1,2,3*^, and Alexander Revzin^1*^

^1^ Department of Physiology and Biomedical Engineering, Mayo Clinic, Rochester, MN, USA

^2^ Enteric Neuroscience Program (ENSP), Mayo Clinic, Rochester, MN, USA

^3^ Division of Gastroenterology and Hepatology, Department of Medicine, Mayo Clinic, Rochester, MN, USA

*Corresponding authors: [revzin.alexander@mayo.edu](mailto:revzin.alexander@mayo.edu); [beyder.arthur@mayo.edu](mailto:beyder.arthur@mayo.edu); [linden.david@mayo.edu](mailto:linden.david@mayo.edu); [druliner.brooke@mayo.edu](mailto:druliner.brooke@mayo.edu)

**Supplementary Information**


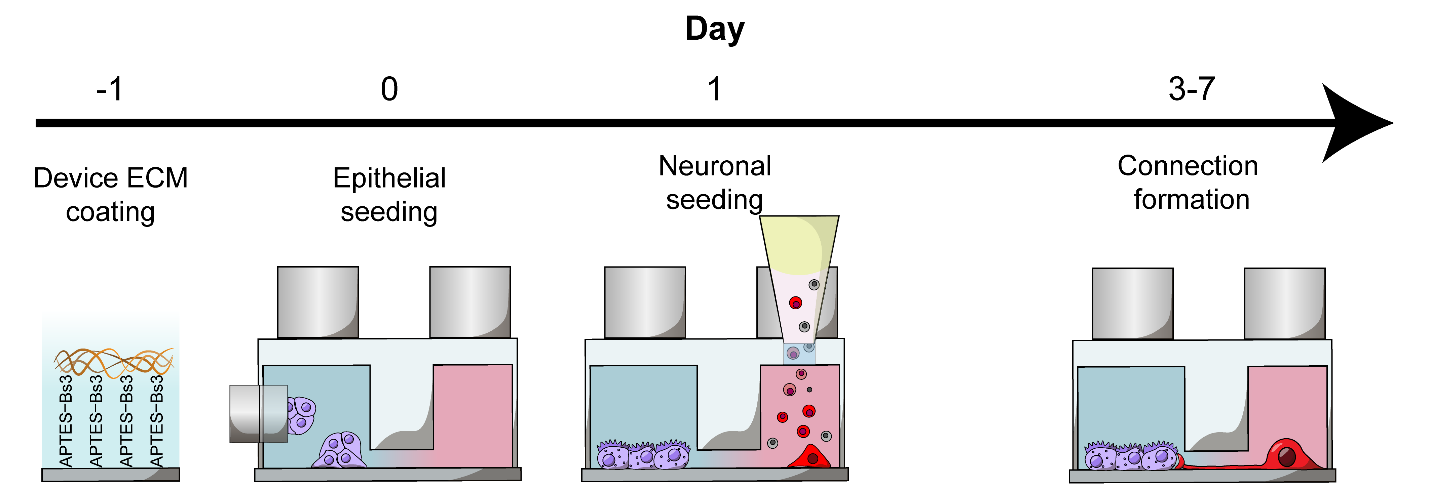


**Figure S1:** **Illustration of cell seeding steps to create neuro-epithelial co-cultures.** Microfluidic devices were coated with cell-adhesive ligands and UV-sterilized prior to cell seeding. Dissociated colon organoids were injected into the epithelial compartment using the side injection port and were given 24 h to attach and begin planarization process. On the next day, neuronal cells were seeded into the adjacent compartment. Neuronal processes extended into epithelial compartment with first neuro-epithelial connections observed at day 3 of culture. Cells were fixed after 3-7 days for culture for morphological analyses.

**Supplement Video 1:** Formation of neuro-epithelial connections in microfluidic cultures.
